# Short-term effect after soft tissue manipulation session on subjective and objective parameters in office workers with chronic low back pain: A randomized clinical trial

**DOI:** 10.1371/journal.pone.0336685

**Published:** 2025-11-21

**Authors:** Michał Wendt, Jakub Rubach, Małgorzata Waszak

**Affiliations:** Department of Medical Biology, Poznan University of Physical Education, Poznan, Poland; Nishikyushu University: Nishikyushu Daigaku, JAPAN

## Abstract

**Background:**

The very frequent occurrence of low back pain (LBP) among office workers forces the search for effective therapeutic tools. The aim of this study was to assess the effectiveness of soft tissue manipulation (Deep Tissue Massage – DTM) on subjective and objective parameters in a group of office workers with chronic low back pain (CLBP).

**Materials and methods:**

Forty participants aged 30–60 years with CLBP were randomly assigned to an experimental group (DTM, N = 20) or a control group (no therapy, N = 20). The intervention included four DTM sessions (45 minutes each over 2 weeks). Outcomes measured were lumbar range of motion (goniometer), pressure pain threshold of the longissimus muscle (algometer), and pain intensity (VAS). Assessments were performed one day prior to and one day following the therapy.

**Results:**

DTM significantly improved lumbar spine mobility in the experimental group across all movement planes (Cohen’s d = 1.31–1.90), with significant between-group differences only for lumbar posterior flexion (p = 0.0149). A significant time effect was also observed for PPT, with an increase in the right-side threshold (p = 0.0186; d = 0.58). Pain intensity decreased significantly in the experimental group for maximum pain during the last week and during sitting (>30 min) (p ≤ 0.01; r = 0.79–0.90). No significant changes were observed in the control group, although a trend toward lower pain levels was noted in the experimental group after intervention.

**Conclusions:**

Four DTM treatments resulted in reduced pain and increased spinal mobility in office workers suffering from chronic low back pain. However, further studies with a larger number of participants and an assessment of long-term effects are necessary.

**Clinical Trial Registration:**

ClinicalTrials.gov NCT05690178

## 1. Introduction

Low back pain (LBP) is the most common form of musculoskeletal disorder [1]. It is defined by the location of the pain, usually between the edges of the lower ribs and the buttock creases [2]. Pain may be centrally located, unilateral or bilateral [3]. There may also be accompanying pain in the leg/legs or neurological symptoms (sensory and motor deficits of the lower limbs) [2]. Disorders of the intervertebral discs [4], intervertebral joints [5] and vertebral endplates (modic changes) [6] are thought to be responsible for most cases of LBP. There are also several serious causes of LBP such as vertebral fractures, malignancy, infection or inflammatory disorders [7]. These occur much less often and require additional diagnostics and specific therapy. Scientific research shows that the muscle tone and stiffness of the lumbar extensor muscles are much higher in patients with chronic low back pain (CLBP) than in healthy people, while the elasticity is much lower [8]. If symptoms of LBP persist for three months or more, the condition is classified as chronic low back pain [9]. It leads to spinal mobility disorders and long-term limitation of the patient’s functioning [3]. This broadly understood pain syndrome is referred to as a multidimensional dysfunction. It can affect the functioning of the patient not only in the physical sphere, but also mentally, professionally and socially [10]. LBP is now the leading cause of disability worldwide [11]. Nearly 75% of office workers experience back pain either acutely or chronically [12]. The global burden of LBP is projected to increase even more in the coming decades, especially in low- and middle-income countries [11].

The basic goals of rehabilitation of patients with CLBP include the elimination of pain, restoring the lost range of motion of the spine, increasing the strength of the stabilising muscles of the trunk and overall improvement of functioning [13]. This can be achieved through various exercise protocols, manual therapy, various forms of massage, relaxation techniques, various soft tissue therapies and re-education of activities of daily living. Although there are numerous scientific studies on the effectiveness of various therapeutic methods in the treatment of LBP, the evidence of their effectiveness is highly obscure [14–19].

Deep tissue massage (DTM) belongs to a broadly understood group of therapeutic methods described as soft tissue manipulation. It is defined as the manual action of a therapist in the superficial and deep layers of muscles and fascia to relax, change abnormal patterns, eliminate tension and pain in the most ergonomic way [20]. DTM should be defined as a stand-alone therapy method using a specific set of techniques and principles. It is a safe form of massage with a wide range of applications, the techniques of which can be modified and improved to meet the needs of the patient. It is believed that DTM can reduce muscle tension, stretch muscles and fascia, improve joint range of motion, improve tissue blood supply, reduce pain and improve the overall function of the musculoskeletal system [21].

The aim of this study was to assess the effectiveness of DTM on subjective and objective parameters in a group of office workers with CLBP. In the scientific literature known to the authors, there are no studies evaluating the effectiveness of DTM in the context of changes in the parameters studied in a group of office workers with CLBP. The conducted experiment is therefore innovative. The authors of the study hypothesized that DTM would increase all ranges of motion of the lumbar spine, increase the first discomfort threshold of the longissimus muscle and reduce back pain in the office workers group with CLBP.

## 2. Materials and methods

**Fig 1:**
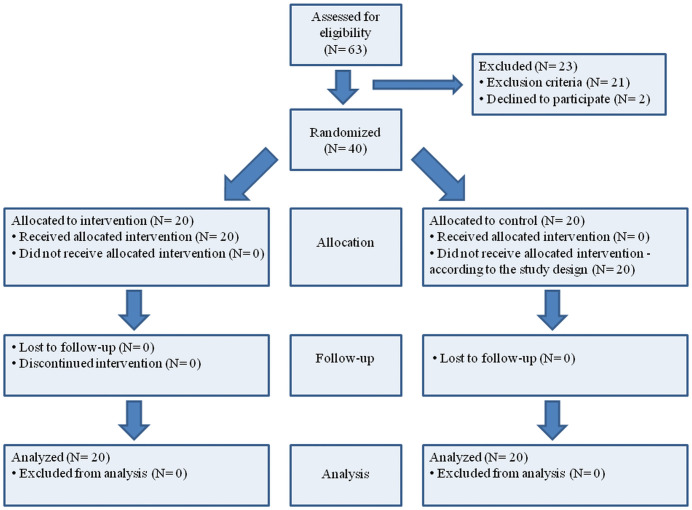
Flow of participants through all stages of the study.

The study entitled “The effect of soft tissue manipulation on subjective and objective parameters in office workers with chronic low back pain: a randomized clinical trial” was a randomized clinical trial with a parallel design. Simple randomization was used using a table of random numbers. All measurements (PRE and POST) were carried out in the same place: the City Hall in Wolsztyn (Poland). For the duration of the study, we were provided with a room that we could use as a research room. This room had a constant temperature (24 degrees Celsius) and was air-conditioned. The study was double-blinded (researcher, outcome assessor). Allocation concealment mechanizm: Random selection of even and odd numbers from the set of 40. Even numbers indicated belonging to the experimental group to which the DTM was applied. Odd numbers indicated belonging to the control group. Participants did not know which study group they belonged to. Each participant drew a number that was characteristic of a given research group (experimental DTM group or control group). The researcher (person performing measurements) also did not know which group the participant belonged to (he only used the participant number). Outcome assessor did not know which group was experimental and which was control (they were marked with numbers instead of names). The coordinator was an independent person who prepared the set of numbers necessary for the allocation of participants. This person ensured that this information was kept hidden from the rest of the research team and the participants. The coordinator supervised the proper conduct of the study. The recruitment period for participants was from 02/08/2021–31/08/2021. The observation period was from 1/09/2021, to 20/10/2021. The study proceeded as planned and was completed after reaching the required sample size. The authors used CONSORT reporting guidelines [22]. A detailed description of the flow of participants through all phases of the study is presented in Fig 1.

### 2.1. Participants

A total of 40 individuals (30–60 years of age) participated in the study. All participants were characterised by the presence of CLBP syndrome, performed office work for eight hours a day and were characterised by moderate physical activity (eligibility criteria). The exclusion criteria included: sciatica, neurological symptoms of the lower limbs (sensory and motor disorders), osteoporosis, lumbar spine surgery, cancer, rheumatic diseases, cauda equina syndrome, pregnancy, while receiving another CLBP therapy during the research study period. Using simple randomisation (table of random numbers), participants were divided into two groups (experimental and control). The experimental group (E) included participants who received DTM (N = 20, 18 women and 2 men, average age: 40 years). The control group (C) included participants who did not receive therapy (N = 20, 13 women and 7 men, average age: 43 years). Detailed characteristics of the participants are presented in Table 1.

**Table 1:** Characteristics of the study participants.

Parameter	Experimental group N = 20				Control group N = 20			
	Mean	SD	MIN	MAX	Mean	SD	MIN	MAX
Body height [cm]	165.35	5,51	157	176	170.4	7.69	156	181
Body weight [kg]	68.46	12.45	55	96	70.95	10.31	55	93
BMI [kg/m^2^]	24.98	3.9	20.2	34.09	24.41	2.83	19.61	29.35
Age [years]	42.05	7.48	30	59	44.35	9.49	30	59
LBP duration (years)	3.5	2.73	0.5	10	4.07	2.84	0.5	9
Office work duration (years)	13.6	5.51	6	24	14.6	7.3	5	25
	Number of patients in the group							
	Experimental group N = 20				Control group N = 20			
Male sex	2				7			
Female sex	18				13			
Pain area								
Spine	13				14			
Buttock	5				5			
Thigh	2				1			
Pain side								
Right	8				9			
Left	9				7			
Central	3				4			

### 2.2. Intervention

The DTM lasted 45 minutes. The entire therapy included four treatments over a period of two weeks. The interval between treatments was three days. DTM was performed by a physiotherapist with 5 years of experience.

The conducted therapy included techniques for: quadratus lumborum muscle, erector spinae muscle, thoracolumbar fascia, ilicac muscle. All techniques were performed on both sides of the patient’s body. Therapeutic techniques were performed based on the Riggs methodology [20].

Technique for the quadratus lumborum and lumbar erector spinae – the patient was in a prone position, with the therapist standing on the side opposite the muscle group being worked on. The technique was performed using the ball of the thumb and the ball of the little finger. The therapist pushed the muscles in a slow lateral movement.

Technique for the lumbar erector spinae – the patient was in a prone position, with the therapist standing on the lateral side of the muscle group being worked on. Using the knuckles and fists the therapist moved the skin and muscle tissue along the lumbar erector spinae.

Technique for the lumbar erector spinae and thoracolumbar fascia – the patient was in a prone position, with the therapist standing on the side opposite the muscle group being worked on. The technique was performed using the therapist’s hands (with the upper limbs crossed). The therapist then moved them apart, exerting a stretching effect on the thoracolumbar fascia and the underlying muscles.

The technique for the iliopsoas muscle involved the patient lying on their back with the lower limb in a triflexion position (on the lateral side of the muscle being worked on). The therapist stood on the same side. With one hand, the therapist stabilized the lower limb and with the other palpated the iliac or psoas muscle on the same side. After locating them, the therapist pressed the iliac and then the psoas muscle with the fingertips.

The physiotherapist performed each technique depending on the participant’s soft tissue response and symptoms.

### 2.3. Research methods

#### 2.3.1. Electrogoniometer.

A Penny & Giles tensometric electrogoniometer with two sensors (SG150 – two-axis, Q110 – single-axis) was used. Measurements were taken in a standing position, in accordance with the measurement methodology according to Lewandowsky [23]. All movements of the lumbar spine (anterior flexion, posterior flexion, right flexion, left flexion, right rotation, left rotation) were examined. The average of three measurements (angular range of motion) was the result of movability for a given direction. The sensors were attached to the skin using Biometrics double-sided tape. The upper sensor (lower edge of the sensor) was placed on the T12 spinous process, while the lower sensor (upper edge) was mounted on the base of the sacrum. The Penny & Giles tensometric electrogoniometer is a reliable and repeatable scientific apparatus for measuring segmental spinal mobility [23].

#### 2.3.2. Algometer.

A Wagner Instruments algometer was used to assess the subjective parameter, i.e., the first discomfort threshold (kg/cm^2^). In the study, the measurement site was a point located on the longissimus muscle, which is a component of the erector spinae muscle. The subjects lay on their front. The pressure, detected by the algometer sensor, was applied from above and perpendicularly to the examined muscle (two fingers laterally from the L1 spinous process). Three measurements were made alternately for both sides of the examined muscle. From these measurements, mean values were calculated, which were the results for the right and left sides of the examined muscle. The algometer test is a reliable and reproducible method of assessing the threshold of discomfort and pain of varying intensity [24].

#### 2.3.3. Visual analogue scale (VAS).

This subjective tool is used to assess the level of pain. The participant marks the level of pain on a 10 cm scale, where zero is no pain and ten is the maximum pain they have ever felt. Current pain (VAS1), last week maximal pain (VAS2) and last week maximal sitting pain (> 30 min) (VAS3) were measured using this subjective tool. The VAS is a reliable research tool to assess the level of pain in LBP [25]. The determination of the Minimal Clinically Important Difference (MCID) for the VAS depends on the type of pain, the underlying condition, and the method used for its estimation [26–30].

In order to assess the impact of DTM therapy on the study population, two measurements were made over time: 1) the day before first intervention; and 2) the day after last intervention.

### 2.4. Statistical methods

The sample size was calculated a priori using G*Power 3.1.9.7 for a repeated-measures ANOVA (within–between interaction; α = 0.05, power = 0.90, assumed effect size η² = 0.30, converted to Cohen’s f = 0.655). The required sample was N = 32 (16 per group); allowing for a 20% dropout rate, the target sample was increased to N = 40 (20 per group). The main outcome variable used in the calculations was pain intensity measured using the VAS scale. The effect size was set at f = 0.30, which corresponds to a moderate/large effect according to Cohen’s conventions. This choice was based on previous studies that demonstrated clinically significant reductions in pain and improvements in function following manual therapies and soft tissue techniques in patients with chronic low back pain [31–33]. In the absence of precise estimates for DTM in office workers, this value was considered a conservative and appropriate assumption for sample size calculation.

All statistical analyses were performed using Statistica 13.3 (StatSoft, USA). The normality of data distribution was verified using the Shapiro–Wilk test, and the homogeneity of variances was assessed with Levene’s test. Depending on data distribution and measurement level, parametric or nonparametric tests were applied.

To assess the impact of DTM therapy on the analyzed variables, a repeated-measures ANOVA (PRE and POST) was applied. This model allowed evaluation of the main effects of time and group, as well as the time × group interaction.

When a significant interaction effect was found, post-hoc analyses were performed to examine simple effects. Paired t-tests were used for within-group comparisons (PRE vs. POST) in both the experimental (E) and control (C) groups, and independent t-tests were used for between-group comparisons (E vs. C) at baseline (PRE) and after the intervention (POST). For non-normally distributed variables, corresponding nonparametric tests were used — the Wilcoxon signed-rank test for within-group and the Mann–Whitney U test for between-group comparisons.

To control for multiple comparisons, the Bonferroni correction was applied within each family of tests. Since two comparisons were made within each family, the adjusted significance level was set at α′ = 0.05/2 = 0.025.

Effect sizes were calculated for all relevant tests: Cohen’s d for t-tests and r for nonparametric tests (r = Z/√N). For ANOVA results, partial eta squared (η²) was reported as a measure of effect size.

The interpretation of effect sizes followed Cohen’s (1988) guidelines:

- η² ≈ 0.01 – small, η² ≈ 0.06 – medium, η² ≥ 0.14 – large;
- d ≈ 0.20 – small, d ≈ 0.50 – medium, d ≥ 0.80 – large;
- r ≈ 0.10 – small, r ≈ 0.30 – medium, r ≥ 0.50 – large.

### 2.5. Ethics

All measurement methods used were non-invasive and safe for health. Written informed consent of the participants and the approval of the Bioethics Committee at the Poznan University of Medical Sciences (Approval Number: 270/21) were obtained. Research have been performed in accordance with the Declaration of Helsinki. The relevant guidelines and regulations of the local institute were strictly followed when conducting the study. The study was registered in the clinical trials registry ClinicalTrials.gov (ID: NCT05690178, date of registration: 19/01/2023).

## 3. Results

### 3.1. The influence of DTM on the objective parameters of spine functioning (goniometric parameters) in people with CLBP

In order to determine the impact of the applied DTM therapy on the values of goniometric parameters, the angular values of the range of motion in the lumbar spine were compared between the experimental group (E) – in which the therapy was performed, and the control group (C) – without therapy, in two time periods: before (PRE) and after (POST) therapy. Analysing the results of the analysis of variance for repeated measurements (in which the factor was the group and measurements repeated over time), a statistically significant difference was noted between the measurements (PRE and POST) for all tested goniometric features of the lumbar spine and a significant impact of the interaction effect between the group and the measurement for movements in the sagittal and frontal planes (Table 2). In the analysis of variance (ANOVA), in addition to significance levels (p), the effect size measure partial eta squared (η²) was also reported. The η² value represents the proportion of variance in the dependent variable explained by a given effect, after accounting for the influence of other factors and random error. Interpretation of effect size followed the thresholds proposed by Cohen (1988), which are commonly used in ANOVA analyses: η² ≈ 0.01 – small effect, η² ≈ 0.06 – medium effect, η² ≥ 0.14 – large effect.

**Table 2:** Summary of the repeated-measures ANOVA analysis for goniometric parameters.

Variable	Group (E, C)		RM (PRE, POST)		RM*Group	
	p	η^2^	p	η^2^	p	η^2^
LAF	0.5852	0.0079	<0.0001**	0.5155	0.0003**	0.3675
LPF	0.1971	0.0433	<0.0001** **	0.5053	<0.0001**	0.3929
LRF	0.3380	0.0242	<0.0001**	0.4123	0.0194*	0.1356
LLF	0.9681	0.0000	<0.0001**	0.5399	0.0001**	0.3522
LRR	0.0702	0.0837	0.0001**	0.3330	0.1935	0.0441
LLR	0.2209	0.0392	0.0002**	0.3104	0.3000	0.0282

LAF – lumbar anterior flexion; LPF – lumbar posterior flexion; LRF – lumbar right flexion; LLF – lumbar left flexion; LRR – lumbar right rotation; LLR – lumbar left rotation; E – experimental group; C – control group; RM (PRE, POST) – measurement before and after therapy; RM*Group – interaction between measurements (PRE and POST) and factor group (E, C); p – test probability; ** – significant differences at the level of α ≤ 0.01; * – significant differences at the level of α ≤ 0.05; η2 – partial eta-square; η² ≈ 0.01 – small effect, η² ≈ 0.06 – medium effect, η² ≥ 0.14 – large effect.

The obtained partial eta squared coefficients revealed a large effect of time (RM: PRE, POST) and a substantial time × group interaction (RM × Group) for the range of motion in the sagittal and frontal planes (Table 2).

**Fig 2:**
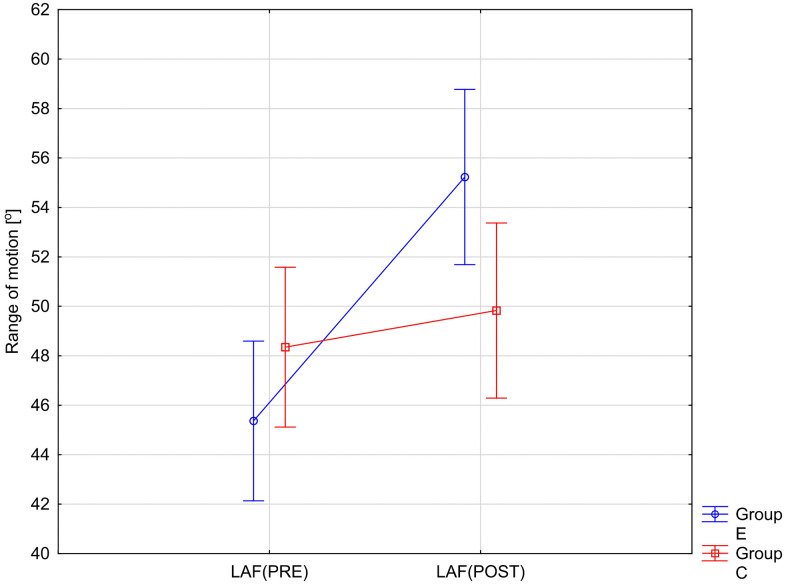
ANOVA interaction group x time (p = 0.0003). Mean angular values of the range of motion: lumbar anterior flexion (LAF) before therapy (PRE), after therapy (POST) in the experimental group (E) and in the control group **(C)**.

**Fig 3:**
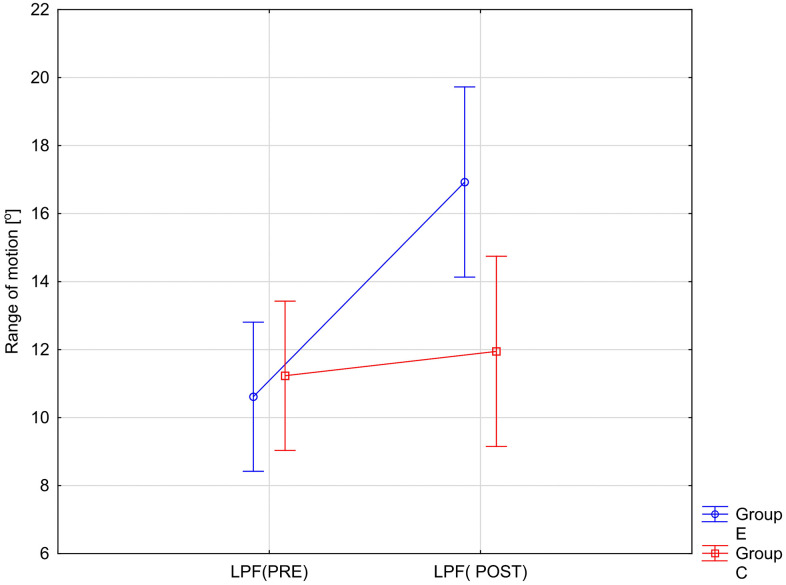
ANOVA interaction group x time (p < 0.0001). Mean angular values of the range of motion: lumbar posterior flexion (LPF) before therapy (PRE), after therapy (POST) in the experimental group (E) and in the control group **(C)**.

**Fig 4:**
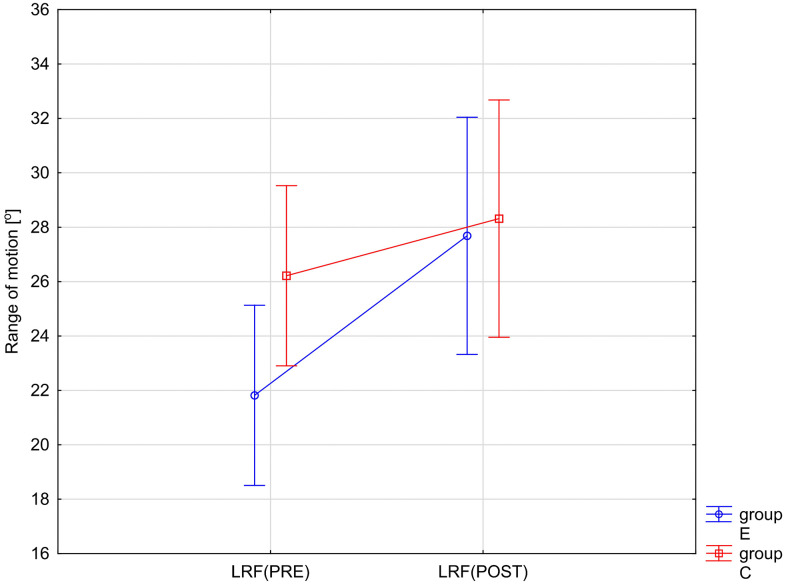
ANOVA interaction group x time (p = 0.0194). Mean angular values of the range of motion: lumbar right flexion (LRF) before therapy (PRE), after therapy (POST) in the experimental group (E) and in the control group **(C)**.

**Fig 5:**
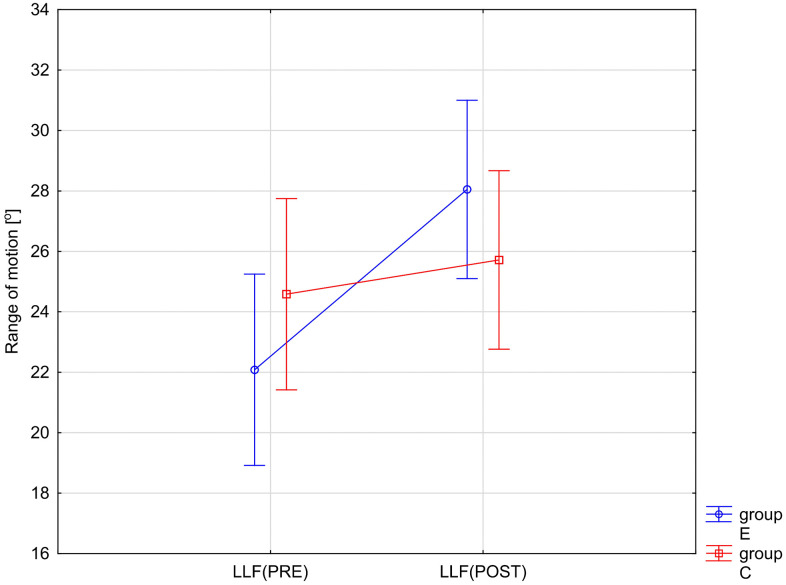
ANOVA interaction group x time (p = 0.0001). Mean angular values of the range of motion: lumbar left flexion (LLF) before therapy (PRE), after therapy (POST) in the experimental group (E) and in the control group **(C)**.

The variables showing a statistically significant RM × Group interaction effect are illustrated in Figs 2–5. The analysis of the presented graphical images shows that the differences in the values of all goniometric parameters between the first and second examinations were significantly greater in the experimental group, i.e., in the patients undergoing therapy, than in the control group.

Following a significant group×time interaction in the repeated-measures ANOVA, we performed simple-effects post-hoc tests. Paired t-tests were used for within-group comparisons (PRE vs POST) in both the control (C) and experimental (E) groups, and independent t-tests were used for between-group comparisons (E vs C) at baseline (PRE) and after intervention (POST). Within-group (2 tests) and between-group (2 tests) comparisons were treated as separate families and Bonferroni correction was applied within each family (α′ = 0.05/2 = 0.025). The results of these analyses are presented in Table 3.

**Table 3:** Within- and between-group comparisons of goniometric measurements (PRE vs. POST) — t-test results (Bonferroni per-outcome correction) and Cohen’s d effect sizes.

Variable	Group/Time	X ± SD	Comparison	t	p	Cohen’s d
LAF [°]	C (PRE)	48.35 ± 7.01	C: PRE–POST	1.74^a^	0.0971	0.39
	C (POST)	49.83 ± 7.58	E: PRE–POST	6.28^a^	<0.0001**	1.41
	E (PRE)	45.36 ± 7.26	E vs C (PRE)	−1.32^b^	0.1939	−0.42
	E (POST)	55.23 ± 8.07	E vs C (POST)	2.18^b^	0.0354	0.69
LPF [°]	C (PRE)	11.23 ± 4.38	C: PRE–POST	0.91^a^	0.0655	0.20
	C (POST)	11.94 ± 5.22	E: PRE–POST	5.86^a^	<0.0001**	1.31
	E (PRE)	10.61 ± 5.27	E vs C (PRE)	−0.4^b^	0.6897	−0.13
	E (POST)	16.93 ± 7.01	E vs C (POST)	2.55^b^	0.0149*	0.81
LRF [°]	C (PRE)	26.22 ± 9.18	C: PRE–POST	1.64^a^	0.1169	0.37
	C (POST)	28.32 ± 12.13	E: PRE–POST	6.79^a^	<0.0001**	1.52
	E (PRE)	21.82 ± 4.78	E vs C (PRE)	−1.90^b^	0.0648	−0.60
	E (POST)	27.68 ± 6.22	E vs C (POST)	−0.21^b^	0.8365	−0.07
LLF [°]	C (PRE)	24.58 ± 8.25	C: PRE–POST	1.05^a^	0.1139	0.23
	C (POST)	25.72 ± 7.90	E: PRE–POST	6.11^a^	<0.0001**	1.37
	E (PRE)	22.08 ± 5.45	E vs C (PRE)	−1.35^b^	0.2652	−0.43
	E (POST)	28.05 ± 4.76	E vs C (POST)	1.13^b^	0.2650	0.36
LRR [°]	C (PRE)	7.35 ± 3.33	C: PRE–POST	1.61^a^	0.1242	0.36
	C (POST)	9.85 ± 7.24	E: PRE–POST	8.47^a^	<0.0001**	1.90
	E (PRE)	8.52 ± 2.82	E vs C (PRE)	1.20^b^	0.2387	0.38
	E (POST)	13.20 ± 3.81	E vs C (POST)	1.83^b^	0.0750	0.58
LLR [°]	C (PRE)	7.27 ± 3.08	C: PRE–POST	1.45^a^	0.0707	0.32
	C (POST)	9.57 ± 6.13	E: PRE–POST	6.24^a^	0.0003**	1.40
	E (PRE)	7.85 ± 3.26	E vs C (PRE)	0.75^b^	0.5641	0.22
	E (POST)	11.72 ± 3.57	E vs C (POST)	2.04^b^	0.1834	0.62

LAF – lumbar anterior flexion; LPF – lumbar posterior flexion; LRF – lumbar right flexion; LLF – lumbar left flexion; LRR – lumbar right rotation; LLR – lumbar left rotation; C – control group; E – experimental group; PRE – before intervention; POST – after intervention; X – mean; SD – standard deviation; ^a^ – paired t-test; ^b^ – independent t-test; * – Bonferroni-adjusted significance level α′ = 0.05/2 = 0.025; ** – Bonferroni-adjusted significance level α′ = 0.01/2 = 0.005; effect size interpretation (Cohen’s d): negligible < 0.20; small = 0.20–0.49; medium = 0.50–0.79; large ≥ 0.80

Post-hoc analyses with Bonferroni correction revealed significant improvements in the experimental group (E) between PRE and POST measurements across all six goniometric variables (all p < 0.0001). Effect sizes were large to very large (Cohen’s d = 1.31–1.90), indicating strong intervention effects (Table 3). In contrast, no significant PRE–POST changes were observed in the control group (C) (all p > .05), with only small effect sizes (d = 0.20–0.37) (Table 3). Between-group comparisons showed no significant differences at baseline (all p > .05). After the intervention, differences between groups increased, particularly in LAF and LPF, which exhibited medium effect sizes (d ≈ 0.80). However, after applying the Bonferroni correction, these differences remained significant only for LPF (p = 0.0149) (Table 3).

In summary, the intervention produced robust improvements in all goniometric outcomes in the experimental group, while no significant changes were detected in the control group.

### 3.2. The impact of DTM on subjective parameters regarding the functioning of the lumbar spine (PPT and pain level) in people with CLBP

Another aim of this study was to determine the effect of the therapy on the subjective parameter, i.e., the pressure pain threshold (PPT) of the right and left longissimus muscles. To achieve it, this parameter was measured both in the experimental group before therapy (PRE) and after therapy (POST) and in the control group at the same time interval. Following the results of the analysis of variance for repeated measures (in which the factor was the group and repeated measurements over time), a statistically significant difference in the value of the threshold of first discomfort was observed between the first and second measurements (PRE and POST) on both the right and left longissimus muscles (Table 4). However, there was no significant differentiation of the examined subjective parameters depending on the group of patients and the interaction between the group and repeated measurements over time (Table 4). The obtained partial eta squared coefficients revealed a medium effect of time (RM: PRE, POST) for PPT-R and PPT-L (Table 4).

**Table 4:** Summary of the repeated-measures ANOVA analysis for the subjective characteristic defining the feeling of first discomfort on pressure of the right and left longissimus muscles (PPT-R and PTT-L).

Variable	Group (E, C)		RM (PRE, POST)		RM*Group	
	p	η^2^	p	η^2^	p	η^2^
PPT-R	0.6611	0.0053	0.0214*	0.1350	0.1993	0.0441
PPT-L	0.6595	0.0052	0.0344*	0.1124	0.3592	0.0222

PPT-R – the threshold of the first discomfort on the pressure of the right longissimus muscle; PPT-L – the threshold of the first discomfort on the pressure of the left longissimus muscle; E – experimental group; C – control group; RM (PRE, POST) – measurement before and after therapy; RM*Group – interaction between measurements (PRE and POST) and factor group (E, C); p – test probability; * – significant differences at the level of α ≤ 0.05; η2 – partial eta-square; η² ≈ 0.01 – small effect, η² ≈ 0.06 – medium effect, η² ≥ 0.14 – large effect.

A significant main effect of time (PRE, POST) on PPT variables prompted within-group comparisons (PRE vs POST) in both the experimental (E) and control (C) groups using the paired t-test with Bonferroni correction. In the experimental group (E), a significant increase in the pressure pain threshold on the right side (PPT-R) was observed after the intervention (t = 2.57; p = 0.0186; d = 0.58). On the left side (PPT-L), an increase in threshold values was also noted (t = 2.05; p = 0.0549; d = 0.46); however, this change did not reach statistical significance after applying the Bonferroni correction (α′ = 0.025) (Table 5). No significant changes in PPT values were found in the control group (C) (Table 5).

**Table 5:** Within- and between-group comparisons of the first discomfort threshold (PPT) before and after the intervention (PRE vs. POST) — results of t-tests (Bonferroni per-outcome correction, α′ = 0.05/2 = 0.025) and Cohen’s d effect sizes.

Variable	Group/Time	X ± SD	Comparison	t	p	Cohen’s d
PPT-R [kg/m^2^]	C (PRE)	5.11 ± 2.71	C: PRE–POST	0.556 ^a^	0.4377	0.124
	C (POST)	5.31 ± 2.42	E: PRE–POST	2.574 ^a^	0.0186*	0.576
	E (PRE)	4.91 ± 2.14	E vs C (PRE)	−0.259 ^b^	0.7931	−0.082
	E (POST)	5.90 ± 2.67	E vs C (POST)	0.732 ^b^	0.4716	0.232
PPT-L [kg/m^2^]	C (PRE)	4.99 ± 2.54	C: PRE–POST	0.940 ^a^	0.3386	0.210
	C (POST)	5.28 ± 2.30	E: PRE–POST	2.045 ^a^	0.0553	0.457
	E (PRE)	5.09 ± 2.26	E vs C (PRE)	0.132 ^b^	0.8893	0.042
	E (POST)	5.84 ± 2.72	E vs C (POST)	0.703 ^b^	0.4830	0.222

PPT-R – the threshold of first discomfort on the pressure of the right longissimus muscle; PPT-L – the threshold of feeling the first discomfort on the pressure of the left longissimus muscle; PRE – measurement before therapy; POST – measurement after therapy; X – mean; SD – standard deviation; ^a^ – paired t-test; ^b^ – independent t-test; * – Bonferroni-adjusted significance level α′ = 0.05/2 = 0.025; effect size interpretation (Cohen’s d): negligible < 0.20; small = 0.20–0.49; medium = 0.50–0.79; large ≥ 0.80

Between-group comparisons (experimental vs. control), conducted both before the intervention (PRE) and after its completion (POST), revealed no significant differences (p > 0.05) (Table 5), thereby confirming the results of the ANOVA analysis.

Another subjective measure of the therapeutic effect was the assessment of pain intensity using the Visual Analogue Scale (VAS), which is the most commonly used tool for subjective pain evaluation.

The results of the repeated-measures analysis of variance (with group and time as factors) revealed statistically significant differences between the first and second measurements of VAS scores for current pain intensity (VAS1), maximum pain during the past week (VAS2), and maximum pain during sitting (>30 min) over the past week (VAS3) (Table 6).

**Table 6:** Summary of the repeated-measures ANOVA analysis for pain VAS values (VAS1, VAS2, VAS3).

Variable	Group (E, C)		RM (PRE, POST)		RM*Group	
	p	η^2^	p	η^2^	p	η^2^
VAS1	0.2237	0.0387	0.0028**	0.2121	0.1785	0.0471
VAS2	0.4548	0.0148	<0.0001**	0.5434	<0.0001**	0.3728
VAS3	0.1102	0.0510	<0.0001**	0.3831	0.0047**	0.1923

VAS1 – current pain level; VAS2 – maximum pain level in the last week; VAS3 – level of maximum pain while sitting (> 30 min) in the last week; E – experimental group; C – control group; RM (PRE, POST) – measurement before and after therapy; RM*Group – interaction between measurements (PRE and POST) and factor group (E, C); p – test probability; ** – significant differences at the level of α ≤ 0.01; η2 – partial eta-square; η² ≈ 0.01 – small effect, η² ≈ 0.06 – medium effect, η² ≥ 0.14 – large effect.

**Fig 6:**
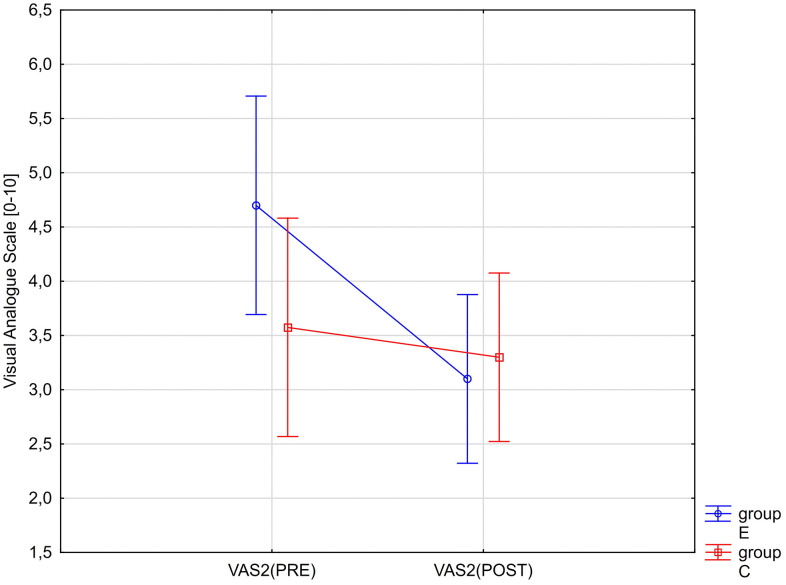
ANOVA interaction group x time (p < 0.0001). Mean values of the maximum pain level in the last week according to the VAS before therapy (PRE) and after therapy (POST) for the experimental (E) and control (C) groups.

**Fig 7:**
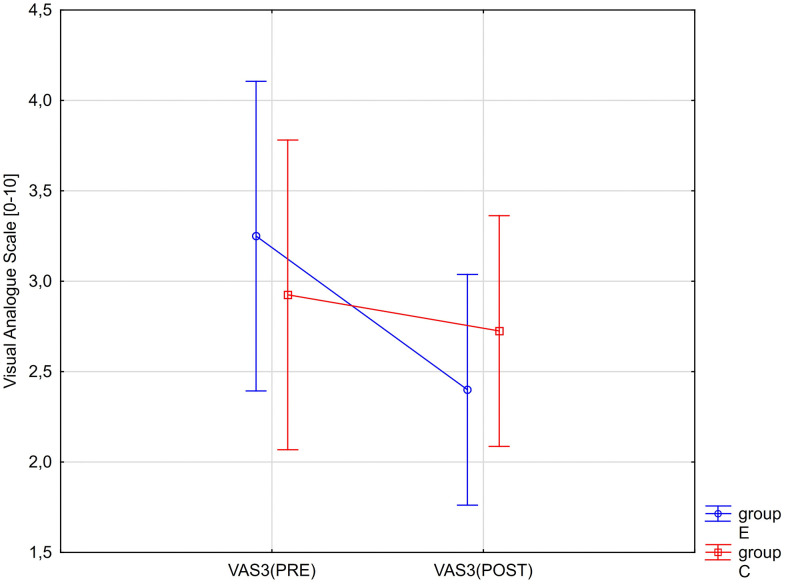
ANOVA interaction group x time (p = 0.0047). Mean values of the level of maximum sensation in the last week of pain while sitting (> 30 min) according to the VAS before therapy (PRE) and after therapy (POST) for the experimental (E) and control (C) groups.

A significant interaction effect between group (E and C) and time (PRE and POST) was also observed for maximum pain intensity and maximum pain during sitting (>30 min). Changes in VAS2 and VAS3 values are presented graphically in Figs 6 and 7.

However, no significant main effect of group on any of the assessed pain perception parameters was found (Table 6). The obtained partial eta-squared values indicate that the time factor (PRE vs. POST) had a strong effect on changes in VAS1, VAS2, and VAS3 values, and that the interaction effect (RM*Group) was also strong for VAS2 and VAS3 (Table 6).

For within-group comparisons of pain intensity levels between the PRE and POST measurements in both groups, the nonparametric Wilcoxon signed-rank test with Bonferroni correction was applied. Statistically significant differences at the level of α ≤ 0.01 were observed only in the experimental group for maximum pain during the last week and maximum pain while sitting (>30 min), whereas a trend toward significance was noted for current pain (α′ = 0.05/2 = 0.025; p for VAS1 = 0.0277) (Table 7). The effect sizes were very large (VAS1: r = 0.90; VAS2: r = 0.88; VAS3: r = 0.79), indicating a strong impact of the applied intervention on pain reduction. In the control group (C), no statistically significant changes were found (all p > 0.05), although large effect sizes (r = 0.83–0.91) were noted, which most likely resulted from the small sample size rather than a true clinical change (Table 7).

**Table 7:** Within- and between-group comparisons of VAS pain scores (VAS1, VAS2, VAS3) before and after the intervention—results of nonparametric tests (Wilcoxon and Mann–Whitney, Bonferroni correction) and effect size (r).

Variable	Group/Time	X ± SD	Comparison	Z	p	r
VAS1	C (PRE)	2.40 ± 1.27	C: PRE–POST	1.82^a^	0.0678	0.91
	C (POST)	2.20 ± 1.28	E: PRE–POST	2.20^a^	0.0277	0.90
	E (PRE)	1.95 ± 2.04	E vs C (PRE)	−1.51^b^	0.1298	0.24
	E (POST)	1.45 ± 1.57	E vs C (POST)	−1.91^b^	0.0505	0.31
VAS2	C (PRE)	3.58 ± 1.80	C: PRE–POST	2.20^a^	0.0779	0.83
	C (POST)	3.30 ± 1.75	E: PRE–POST	3.52^a^	0.0004**	0.88
	E (PRE)	4.70 ± 4.70	E vs C (PRE)	1.18^b^	0.2393	0.19
	E (POST)	3.10 ± 3.10	E vs C (POST)	−0.43^b^	0.6652	0.07
,VAS3	C (PRE)	2.93 ± 1.66	C: PRE–POST	1.82^a^	0.0679	0.91
	C (POST)	2.73 ± 1.43	E: PRE–POST	3.04^a^	0.0024**	0.79
	E (PRE)	3.25 ± 3.25	E vs C (PRE)	0.35^b^	0.7251	0.06
	E (POST)	2.40 ± 2.40	E vs C (POST)	−0.83^b^	0.4094	0.13

VAS1 – current pain level; VAS2 – maximum pain level in the last week; VAS3 – level of maximum pain while sitting (> 30 min) in the last week; PRE – measurement in the first examination; POST – measurement in the second examination; X – mean; SD – standard deviation; Z – standardized test statistic; ^a^ – Wilcoxon signed-rank test; ^b^ – Mann–Whitney U test; p – test probability; ** – Bonferroni-adjusted significance level α′ = 0.01/2 = 0.005; r – correlation coefficient; interpretation of effect size (r): small (r = 0.10–0.29), moderate (r = 0.30–0.49), large (r ≥ 0.50).

Between-group comparisons using the Mann–Whitney U test showed no statistically significant differences between the experimental (E) and control (C) groups at baseline (PRE) for any of the VAS measures (VAS1–VAS3; r = 0.06–0.24), confirming comparable pain levels prior to the intervention (Table 7). Following the intervention (POST), a moderate effect was observed only for current pain intensity (VAS1; r = 0.30); however, this difference did not reach statistical significance after Bonferroni correction, although a trend toward lower pain perception was noted in the experimental group (Table 7).

## 4. Discussion

### 4.1. Impact on objective parameters – active range of motion of the lumbar spine (AROM)

In the conducted study, a statistically significant difference was noted between the measurements (PRE and POST) for all tested goniometric features of the lumbar spine in the experimental group after DTM therapy. There was an average increase in angular values of anterior flexion 9.9° (21.9%), posterior flexion 6.3° (59.7%), right flexion 5.9° (26.9%), left flexion 6.0° (27.0%), right rotation 4.7° (54.9%) and left rotation 3.9° (49.3%). In the control group, there were no significant differences between the PRE and POST tests for any of the examined characteristics. The collected results show a clear improvement in the mobility of the lumbar spine of the participants as a result of DTM application. The observed improvement in spondylometric parameters may be the result of the influence of DTM techniques on myofascial structures. It is believed that by acting on the muscle belly and its tendon elements, it is possible to influence the receptors, e.g., Golgi tendon organs, the subsequent reaction of which is to stretch the muscle, which in turn leads to an increase in ROM [34]. As with other types of myofascial techniques, the effect of increasing the mobility of the musculoskeletal system is probably related to the lengthening of the sarcomeres as a result of manual compression of the hypertonic muscle fibre node [35]. The between-group analysis (POST Experimental group to POST Control group) showed that a statistically significant difference occurred only in relation to posterior flexion, which may indicate that the therapy period was too short.

There is a small amount of research in the scientific literature on the effect of DTM on spinal AROM. There are no studies focusing on the effect of the tested method on the AROM of the lumbar spine. James et al. [36] conducted a study to investigate the effect of Rolfing Structural Integration on the AROM of the cervical spine. This method has some features in common with DTM [21]. The research group consisted of 31 participants with cervical spine dysfunction [36]. A significant increase in left rotation by 13.0°, right rotation by 13.0°, left flexion by 8.0°, and right flexion by 7.0°, anterior flexion by 5.0° and posterior flexion by 5.0° was observed [36]. In our study, we also noted a significant increase in the AROM of the lumbar spine in all planes after the use of DTM in the group of participants with CLBP. Both of these methods are classified as broadly understood soft tissue therapies. Although in both of these experiments a different area of the spine was examined, the combination of these statistical analyses shows that therapies of this type can be used to increase the AROM in people with dysfunctions of the various parts of the spine. Bingölbali et al. [37] came to similar conclusions. In their study, they assessed the effects of DTM on the number of myofascial trigger points (MPS), neck range of motion (ROM), pain, disability and quality of life of patients with Myofascial Pain Syndrome (MPS). The study included participants aged 20–57 years. The control group (N = 40) underwent transcutaneous electrical neuromuscular stimulation (TENS), hot compress and ultrasound. In addition to the above-mentioned physical procedures, the experimental group was additionally administered DTM. A total of 12 procedures were performed in both groups. In the context of changes in ROM parameters, the authors observed a clear improvement in extension, lateral flexion, right and left rotation in the ROM of the neck in the experimental group compared to the control group. Bingölbali et al. [37] concluded that DTM was a key therapeutic element in the group of participants with cervical MPS, responsible for increasing the cervical spondylometric parameters studied. Saíz-Llamosas et al. [38] conducted a study aimed at examining whether the application of the cervical myofascial induction technique aimed at the nuchal ligament caused changes in asymptomatic people, e.g., in AROM. The study involved 35 people, with an average age of 21 + / − 4 years, without pain in the neck, shoulder girdle or arms. Participants were randomly divided into two groups: an experimental group that received the true cervical myofascial induction technique and a control group that received the sham-manual procedure. Saíz-Llamosas et al. [38] concluded that the use of the cervical myofascial induction technique resulted in an increase in flexion, extension and lateral flexion to the left in a group of asymptomatic individuals. There were no significant changes for rotational movements. In the study we conducted, we noted an improvement in all spondylometric parameters of the lumbar spine. Differences in the collected results may result from the selection of the research material. Saíz-Llamosas et al. [38] evaluated the effectiveness of the cervical myofascial induction technique, which like DTM is classified as soft tissue therapy, in a group of young asymptomatic patients. The absence of cervical spine dysfunction in the research group could prevent the capture of the full therapeutic effect of the tested method.

There are several studies in the scientific literature showing the beneficial effect of soft tissue therapy (having many features in common with DTM) on ROM in relation to other anatomical areas. Avrahami & Potvin [39] showed a significant increase in passive hip extension after the use of soft tissue techniques described as fascial-muscular lengthening therapy. These techniques are also used in DTM. Kassolik et al. [40] observed that subjects undergoing tensegrity massage showed statistically significant improvements in the passive and active range of shoulder flexion and abduction. It should be noted that the tested therapy has many features in common with DTM. Forman et al. [41] evaluated the effect of deep stripping massage strokes (DSMS) on hamstring muscle length and strength. The results of their study suggest that DSMS increased hamstring length in less than three minutes, but did not affect the strength parameter.

The authors of this manuscript draw attention to the fact that in the scientific literature on the effectiveness of DTM in the context of changes in spondylometric parameters of the spine, there is a lack of scientific research. There is a justified need to conduct more research in this area in order to better understand the impact of DTM on the AROM of different sections of the spine.

### 4.2. Impact on subjective parameters – PPT and pain level (VAS)

This study reported significant differences in PPT between the first (PRE) and second (POST) measurements, but only in the experimental group with regard to the right longissimus muscle. However, in the control group for this feature, no significant differences were noted between the PRE and POST measurements. The DTM application increased this parameter in the case of the right longissimus muscle from 4.91 to 5.90 kg/cm^2^, and in the case of the left from 5.09 to 5.84 kg/cm^2^. The conducted intragroup analysis showed that DTM had a positive effect on the subjective parameter under study. The increased PPT after DTM application can be attributed to the release of fibrous adhesions. Other mechanisms, such as the central pain-modulating system, are thought to play a role in mediating pain sensation after tissue massage [42]. Nevertheless, the between-group analysis showed no significant differences in this feature between the compared groups, neither in the first (PRE) nor in the second (POST) measurements. The lack of statistical difference between the study groups in the measurement (POST) may be due to the short therapy period. We used four treatments in the entire therapeutic series. We believe that increasing the number of treatments would help show the difference between the study groups in the post-treatment outcome analysis (POST).

The conducted intragroup analyses showed a statistically significant difference between the first (PRE) and second (POST) measurements of the VAS value regarding the maximum pain in the last week (VAS2) and maximum pain while sitting (> 30 min) in the last week (VAS3) as a result of the DTM application. These differences indicate the effectiveness of the DTM analgesic effect in the group of participants with CLBP. In the control group, no significant changes in the examined parameters were noted. The recorded decrease in VAS2 by 1.6 cm and VAS3 by 0.85 cm in the experimental group could be due to the improvement of tissue blood supply in the treated area. The DTM application may have improved cellular metabolism and removed inflammatory chemicals such as prostaglandins, histamine and bradykinin. As a result, it could have had an impact on reducing nociceptor sensitisation [43]. No statistically significant differences were observed between the study groups in terms of VAS changes, which may also indicate that the therapy was too short.

The reported analgesic effects of the tested therapeutic method agree with several scientific articles on similar topics. Zheng et al. [44] evaluated the effectiveness of DTM in a population of patients with non-specific LBP. They reported that DTM and lumbar traction were more effective than lumbar traction alone in increasing PPT (0.4 kg/cm^2^) and reducing pain (VAS – 0.5 mm). The observed changes in subjective parameters were statistically significant [44]. In our study, we did not combine DTM with any other therapy, but we compared the effect with a control group. We noted a greater increase in the PPT index (on the right longissimus muscle an increase of 1 kg/cm^2^, while on the left one by 0.75 kg/cm^2^) and a significant decrease in the level of pain in the experimental group. A study by Zheng et al. [44] shows that DTM can also be combined with other therapeutic methods. Majchrzycki et al. [34] evaluated the effectiveness of DTM versus DTM together with non-steroid anti-inflammatory drugs (NSAIDs) in CLBP. The authors examined 59 participants divided into two research groups in the context of changes in subjective parameters. Majchrzycki et al. [34] reported a significant decrease in pain (measured by the VAS) by 1.6 cm in the DTM and DTM group with NSAIDs. Additionally, in the case of assessing the level of disability measured by the Oswestry and Roland-Morris questionnaires, there was a clear improvement in the participants studied. The lack of statistical difference between the studied groups proves the significant impact of DTM on reducing pain and improving the disability of patients with CLBP [34]. The results obtained in this study confirm the analgesic effect of the tested therapeutic method. Romanowski et al. [31], in 2012, assessed the effectiveness of DTM in a group of people with CLBP. Subjective parameters characterising the level of pain (VAS) and the level of disability measured by the Modified Oswestry Low Back Pain Disability Index (ODI) and the Quebec Back Pain Disability Scale (QBPD) were examined. Romanowski et al. [31] noted a significant improvement in measured subjective parameters, which is consistent with the results of our study. It should be noted that Romanowski et al. [31] studied elderly patients with CLBP (60–75 years). In our study, the research group (patients with CLBP, performing office work) was younger (30–60 years). It can therefore be assumed that DTM may be a therapeutic tool reducing pain in people with CLBP of all ages. Frey Law et al. [45] conducted a double-blind, randomised, controlled trial of the effect of massage on mechanical hyperalgesia and perceived pain using delayed onset muscle soreness (DOMS) as an endogenous model of myalgia. Participants were randomly assigned to a control (no treatment), superficial touch or DTM group. Eccentric wrist extension exercises were performed at visit 1 to induce DOMS 48 hours later at visit 2. Pain was assessed using a VAS and PPT. Measurements were taken at baseline, post-exercise, pre-treatment and post-treatment. DTM reduced pain (48.4% reversal of DOMS) during muscle stretching and mechanical hyperalgesia was also reduced (27.5% reversal) [45]. The authors concluded that DTM can significantly reduce muscle pain symptoms by approximately 25.0–50.0% [45]. In our study, we also noted a significant reduction in subjective parameters. We observed a significant increase in the mean PPT of the longissimus muscles by approximately 17.4% (average of the right and left muscles) due to the DTM application. In the case of pain assessment, we observed an average decrease in pain of approximately 25.6% VAS1 (current pain), 34.0% VAS2 (last week maximum pain) and 26.2% VAS3 (last week maximum pain > 30 min). The statistical analyses performed showed significant improvements in subjective parameters as a result of the DTM application. Slight differences in the compared results may result from the research material. Frey Law et al. [45] studied the effect of DTM on the extensor muscles of the wrist after eccentric exercises. In the case of our study, the research group consisted of people with CLBP, while the PPT measurement itself concerned the longissimus muscle on both sides. In both our study and that performed by Frey Law et al., the improvement in subjective parameters in the DTM group was statistically significant.

### 4.3. Minimal Clinically Important Difference for ROM, VAS, and PPT values

The minimal clinically important difference (MCID) for the Visual Analogue Scale (VAS) is important because it defines the smallest change in score that a patient perceives as a significant improvement or deterioration. This helps clinicians and researchers assess whether a treatment is truly effective and provides a real benefit to the patient, rather than just a statistically significant change.

The generally accepted MCID values for the VAS scale are approximately 1–2 cm per 10 cm of the scale, although they may vary depending on the type of pain, condition, and the method used to determine it. Gallagher et al. [26] defined a change of 13 mm on the VAS scale as the minimum clinically important difference for acute pain. Myles et al. [30] set the MCID at 10 mm in the context of postoperative pain. Ostelo et al. [28], based on a literature review as well as discussions and consultations with experts at the Primary Care Low Back Pain Research Forum, defined a 15 mm change on the VAS scale as the MCID. Hagg et al. [27] in a study of patients with chronic back pain showed that a difference of 18–19 mm on the VAS scale is clinically significant. In their review “Clinically important outcomes in low back pain,” the authors state that for patients with subacute or chronic low back pain, the minimum clinically important change (MCID) on the VAS scale should be at least 20 mm [29].

Therefore, although individual studies provide specific values, the latest scientific data highlight the need for an individual approach to each patient and consideration of the clinical context, rather than relying solely on a single, universal number.

In this study, after four DTM treatments, the experimental group experienced a clinically significant reduction in maximum pain (VAS2) – 16 mm. The reduction in current pain (VAS1) and maximum pain while sitting (VAS3) was not clinically significant.

There is no single, universally accepted MCID for PPT. The literature cites both absolute values (most commonly cited: 1.63 kg/cm² ≈ 160 kPa) and relative thresholds (often given as a range of ~17.0–33.0% change). The value of 1.63 kg/cm² and the percentage range have been proposed/reported in several reviews and articles, but they do not represent a clear consensus — the differences result from different methodologies, measurement locations, populations (MPS, CLBP, healthy), and calculation methods (anchor-based vs. distribution-based) [46–48].

In this study, the changes in PPT in the experimental group recorded after 4 DTM treatments, although statistically significant for PPT-R (p = 0.0186), were not clinically significant, as they were lower than the MCID value most frequently cited in the literature and amounted to 0.99 for PPT-R and 0.75 for PPT-L.

There is also no universally accepted minimal clinically important difference (MCID) for the range of motion of the lumbar spine. The lack of standardized MCID values is primarily due to the high individual variability in range of motion depending on the age, gender, body type, and activity level of the patient. It is difficult to determine a single value that would be relevant for all patients. In addition, the rather diverse methodology for measuring ranges of motion within the spine and the lack of a strong relationship between spinal function and pain symptoms are also significant. For this reason, clinicians assessing a patient’s condition focus more on the patient’s subjective feelings (pain scales, disability questionnaires).

### 4.4. Study limitations and suggestions for future research

This study did not consider a third measurement during the so-called follow-up, which was not possible for organisational reasons. The authors had access to the study group of office workers for a limited time, which made it impossible to perform another follow-up measurement. The results obtained during the next measurement would provide valuable data that would allow for additional analyses and determining whether the obtained effects persist for a longer period of time after the end of therapy.

The authors of the manuscript suggest that there is a great need to study the effectiveness of DTM in the context of changes in the mobility of the spine and in relation to other disorders of the musculoskeletal system. It should be noted that the literature does not consistently support a relationship between spinal range of motion and pain [49]. Therefore, interpreting changes in mobility as a direct reflection of pain reduction should be done with caution. The use of additional measurement tools can help to study the impact of this therapeutic method on other parameters of the musculoskeletal system, e.g., assessment of structural changes or bioelectric potentials of various muscles. Another limitation of this study is that the control group did not receive any therapeutic intervention. Although this allowed for consideration of the natural passage of time over a two-week period, the control group did not reflect placebo effects specific to DTM therapy. In future studies, it would be advisable to include a placebo/sham group or to compare DTM with another active intervention, which would allow for a more precise assessment of the specific and non-specific effects of the therapy.

## 5. Conclusions

1. The use of DTM techniques in the experimental group for four treatments had a positive effect on the mobility of the lumbar spine by a statistically significant increase in the range of all movements occurring in it. The DTM therapy confirms a beneficial effect on the functional parameters of the lumbar spine in people with CLBP.
2. The increase in the PPT value of the right lumbar longissimus muscle after the application of four DTM treatments indicates a beneficial effect of the tested therapy on the PPT in a group of office workers with CLBP.
3. Reduction of pain (measured with the VAS) after DTM application confirms its analgesic therapeutic effect in a group of people with CLBP.
4. The lack of significant statistical changes in the majority of objective and subjective parameters in the between-group analysis may indicate too short DTM therapy period in relation to patients with CLBP. Too few DTM treatments made it impossible to capture a clearer effect in the area of subjective and objective parameters.
5. DTM can be an important therapeutic element in the broadly understood rehabilitation of office workers with CLBP. The authors of the study indicate the need to conduct further research on DTM in the context of its effectiveness in various dysfunctions of the musculoskeletal system, but also in order to identify the mechanisms responsible for the therapeutic effectiveness of the method. We believe that the issues related to the length of therapy and the frequency of individual treatments should be investigated more thoroughly.

## Supporting information

S1 Protocol Trial protocol. (PDF)

S1 Checklist Consort checklist. (PDF)
