# Supplementary material for: Short-term effect after soft tissue manipulation session on subjective and objective parameters in office workers with chronic low back pain: A randomized clinical trial
Source: PLoS One. 2025 Nov 21;20(11):e0336685. doi: 10.1371/journal.pone.0336685 (PMC12637976; doi:10.1371/journal.pone.0336685)
Supplement: S1 Protocol — (PDF) [file pone.0336685.s001.pdf]

Trial protocol

Protocol title: Effect of Soft Tissue Manipulation on Subjective and Objective Parameters in Office Workers With Chronic Low Back Pain: A Randomized Clinical Trial.

Research period: April 2021 – May 2022

Investigators:

1) **Wendt Michał, PhD, PT**
   Poznań University of Physical Education
   Królowej Jadwigi 27/39
   61-871 Poznań, Poland
   Telephone: 61 835 52 26 Ext. +48
   Email: wendt@awf.poznan.pl, wendt.m@interia.pl

2) **Rubach Jakub, MSc, PT**
   Poznań University of Physical Education
   Królowej Jadwigi 27/39
   61-871 Poznań, Poland
   Telephone: 61 835 52 26 Ext. +48
   Email: jakubrubach98@gmail.com

3) **Waszak Małgorzata, PhD**
   Poznań University of Physical Education
   Królowej Jadwigi 27/39
   61-871 Poznań, Poland
   Telephone: 61 835 52 26 Ext. +48
   Email: waszak@awf.poznan.pl

Authors responsibilities:

Study design and conception: M. We., J. R., M. Wa.,
Intervention: J. R.,
Data collection: M. We.,
Statistical analysis: M. Wa.,
Visualization: M. We., M. Wa.,
Writing the manuscript: M. We., M. Wa.,
Revision of the manuscript: M. We., J. R., M. Wa.

Project summary

Background: The aim of the study will be to assess the use of deep child (DTM) on subjective and objective parameters in a group of office workers with chronic low back pain (CLBP).

Material and methods: A total of 40 office workers (aged 30-60) from CLBP will participate in the study. Participants will be divided into two groups: 1) experimental group (these participants will have intervention - Deep Tissue Massage); and 2) a control group (no treatment will be given). The examination will assess the angular values of the mobility of the lumbar spine in all axes (ROM), the pressure pain threshold (PPT) of the longissimus muscles and the level of pain (VAS). Measurements will be made the day before (PRE) and the day after (POST) therapy.

Rationale: In the scientific literature known to the authors, there are no studies evaluating the effectiveness of DTM in the context of changes in the parameters studied in the group of office workers with CLBP. The conducted experiment will therefore be innovative.

Hypothesis: The authors of the study hypothesized that DTM would increase all ranges of motion of the lumbar spine, increase the pressure pain threshold of longissimus muscle, and reduce back pain in a group of office workers with CLBP.

Rationale & background information

The basic goals of rehabilitation of patients with CLBP include the elimination of pain, restoring the lost range of motion of the spine, increasing the strength of the stabilising muscles of the trunk and overall improvement of functioning [1]. This can be achieved through various exercise protocols, manual therapy, various forms of massage, relaxation techniques, various soft tissue therapies and re-education of activities of daily living. Although there are numerous scientific studies on the effectiveness of various therapeutic methods in the treatment of LBP, the evidence of their effectiveness is highly obscure [2-7].

Deep tissue massage (DTM) is defined as the manual action of a therapist in the superficial and deep layers of muscles and fascia to relax, change abnormal patterns, eliminate tension and pain in the most ergonomic way [8]. DTM should be defined as a stand-alone therapy method using a specific set of techniques and principles. It is a safe form of massage with a wide range of applications, the techniques of which can be modified and improved to meet the needs of the patient. It is believed that DTM can reduce muscle tension, stretch muscles and fascia, improve joint range of motion, improve tissue blood supply, reduce pain and improve the overall function of the musculoskeletal system [9]. In the scientific literature, there is a small amount of research confirming the effectiveness of this therapeutic method.

In the scientific literature known to the authors, there are no studies evaluating the effectiveness of DTM in the context of changes in the parameters studied in the group of office workers with CLBP. The conducted experiment will therefore be innovative.

Hypothesis: The authors of the study hypothesized that DTM would increase all ranges of motion of the lumbar spine, increase the pressure pain threshold of longissimus muscle, and reduce back pain in a group of office workers with CLBP.

References

[1] Coole C, Drummond A, Watson PJ, Radford K. What concerns workers with low back pain? Findings of a qualitative study of patients referred for rehabilitation. J Occup Rehabil. 2010;20(4):472-80.

[2] Borges TP, Greve JM, Monteiro AP, et al. Massage application for occupational low back pain in nursing staff. Rev Lat Am Enfermagem. 2012;20:511–9.
[3] Namnaqani FI, Mashabi AS, Yaseen KM, Alshehri MA. The effectiveness of McKenzie method compared to manual therapy for treating chronic low back pain: a systematic review. J Musculoskelet Neuronal Interact. 2019;19(4):492-99.
[4] Gomes-Neto M, Lopes JM, Conceição CS, Araujo A, Brasileiro A, Sousa C, et al. Stabilization exercise compared to general exercises or manual therapy for the management of low back pain: A systematic review and meta-analysis. Phys Ther Sport. 2017;23:136-42.
[5] Thornton JS, Caneiro JP, Hartvigsen J, Ardern CL, Vinther A, Wilkie K, at al. Treating low back pain in athletes: a systematic review with meta-analysis. Br J Sports Med. 2021;55(12):656-62.
[6] Lam OT, Strenger DM, Chan-Fee M, Pham PT, Preuss RA, Robbins SM. Effectiveness of the McKenzie Method of Mechanical Diagnosis and Therapy for Treating Low Back Pain: Literature Review With Meta-analysis. J Orthop Sports Phys Ther. 2018;48(6):476-90.
[7] Last AR, Hulbert K. Chronic low back pain: evaluation and management. Am Fam Physician. 2009;79(12):1067-74.
[8] Riggs A. Deep Tissue Massage: A Visual Guide to Techniques. 1st ed: North Atlantic Books U.S.; 2007.
[9] Koren Y, Kalichman L. Deep tissue massage: What are we talking about? J Bodyw Mov Ther. 2018;22(2):247-51.

Study goals and objectives

The main objective: The main objective of the study will be to assess the impact of deep tissue massage on subjective and objective parameters in a group of office workers with chronic low back pain.

Specific objectives:

- Assessment of the impact of deep tissue massage on the mobility of the lumbar spine in all directions.
- Assessment of the effect of deep tissue massage on the discomfort threshold of tissue compression in the area of the longissimus muscle.
- Assessment of the impact of deep tissue massage on the level of pain.

Study design

The conducted study will be a randomized clinical trial (triple-blind: participants, investigator, evaluator of results) with a parallel design. A simple randomization will be applied using a table of random numbers. All measurements will be carried out in the same place: the City Hall in Wolsztyn. A total of 40 people (aged 30-60) will participate in the study.

Inclusion Criteria:

- CLBP group,
- office work for eight hours a day,
- moderate physical activity (eligibility criteria).

Exclusion criteria:

- sciatica,
- neurological symptoms of the lower limbs (sensory and motor disorders),
- osteoporosis, lumbar spine surgery,
- Cancer,
- rheumatic diseases,
- cauda equina syndrome,
- pregnancy,
- during other CLBP therapy.

Participants will be randomized into two equal groups (experimental and control).

Methodology

Intervention

DTM will last 45 minutes. The entire therapy will include four treatments over a two-week period. The interval between treatments will be three days. The conducted therapy will include techniques for: quadratus lumborum muscle, erector spinae muscle, thoracolumbar fascia, iliopsoas muscle. All techniques will be performed on both sides of the patient's body. DTM will be performed by a physiotherapist with 5 years of experience.

Research methods:

1) **Electrogoniometer**

A Penny & Giles strain gauge electrogoniometer with two sensors (SG150 - two-axis, Q110 - single-axis) will be used. Measurements will be made in a standing position, in accordance with the measurement methodology according to Lewandowski [10]. All movements of the lumbar spine (forward flexion, backward flexion, right flexion, left flexion, right rotation, left rotation) will be tested. The average of the three measurements will be the result of mobility for a given direction. The sensors will be attached to the skin using Biometrics double-sided tape. The upper sensor (lower edge of the sensor) will be placed on the T12 spinous process, while the lower sensor (upper edge) will be placed on the base of the sacrum.

2) **Algometer**

The Wagner Instruments algometer will be used to assess the subjective parameter, i.e. the first threshold of discomfort. The measurement site will be a point located on the longissimus muscle. Participants will be in the front lying position during the measurements. The sensor of the algometer will be applied from above and perpendicularly to the tested muscle (two fingers to the side of the L1 spinous process). Three measurements will be made alternately for both sides of the longissmus muscle under examination. From these measurements, mean values will be calculated, which will be the results for the right and left sides of the tested muscle.

3) **Visual Analog Scale (VAS)**

This subjective tool is used to assess the level of pain. The subject will mark their pain level on a 10-cm scale, where zero is no pain and ten is the maximum pain they have ever felt. Current pain (VAS1), last week maximum pain (VAS2) and last week maximum pain sitting (>30 min) (VAS3) will be measured with this subjective tool.

In order to assess the impact of DTM therapy on the study population, two measurements will be made in time: 1) the day before the therapy; and 2) the day after treatment.

References

[10] Lewandowski J. Formation of Physiological Curvatures and Segmental Mobility of the Human Spine Aged from 3 to 25 Years in Electrogoniometric Studies; AWF Poznań: Poznań, Poland: 2006.

Safety considerations

All planned measurement methods will be non-invasive and safe for health. DTM therapy is also safe and will be performed by an experienced physiotherapist. The authors will seek approval from the local bioethics committee. Experimental procedures will be carried out in accordance with the Declaration of Helsinki. Appropriate guidelines and regulations of the local institute will be strictly followed when conducting the study. The trial will be registered in the ClinicalTrials.gov clinical trials registry. Participants will be able to withdraw from participation at any stage during the study.

Follow-up

Does not apply to this project. It is planned to perform two measurements in time: 1) the day before the therapy and 2) the day after the therapy. No follow up.

Data management and statistical analysis

The adequate sample size was determined using the G*Power 3.1.9 software. The standard threshold $\alpha = .05$ was established; then the acceptable power level of the $1\text{-}\beta = .90$ test and the expected moderate effect size $\eta 2 = .30$ were determined for within-between interaction. The calculated total expected size of the sample is $N = 32$, i.e. 16 subjects in one research group, assuming that their number will not decrease during the study.

The collected data will be included in a database created with Excel 2019-2021, and calculations will be made using the Statistica 12.6 statistical software package (StatSoft. Inc.). Repeated measures ANOVA (PRE and POST) will be used to assess the effect of the DTM on the variables under study. In addition, a partial eta-square ($\eta 2$) will be calculated - the so-called effect size measurement. For intra-group comparisons of the examined features (PRE and POST), the Student's t-test for dependent variables or the non-parametric Wilcoxon test for dependent pairs will be used. On the other hand, for intergroup comparisons, the Student's t-test for independent variables or the non-parametric U Mann-Whitney test for independent pairs will be used.

Quality assurances

The authors assure that they will make every effort to exercise the utmost diligence in the planned scientific research.

Expected outcomes of the study

We believe that the obtained results of the planned study will contribute to a better understanding of DTM effectiveness in the group of office workers with CLBP in the context of changes in subjective and objective parameters. The authors plan to publish their results in a peer-reviewed scientific journal. The results obtained and the conclusions drawn will contribute to the development of broadly understood rehabilitation and improvement of the quality of life of people with CLBP.

Dissemination of results and publication policy

It is planned to publish the research results in a peer-reviewed scientific journal. It is planned that the main researcher (Michał Wendt) will be the first author, the second (Jakub Rubach) and the third (Małgorzata Waszak). Personal data of participants participating in the study will be anonymous.

Duration of the project

It is planned that recruitment will last through August 2021. The period of intervention and collection of results will last through September and October 2021. Data analysis will take place during November and December 2021. At a later stage, it is planned to write a manuscript.

Problems anticipated

No problems are expected that may prevent the planned research project from being carried out.

Ethics

The authors will obtain written informed consent from the participants. The authors will obtain approval from the local bioethics committee. Experimental procedures will be carried out in accordance with the Declaration of Helsinki. Appropriate guidelines and regulations of the local institute will be strictly followed when conducting the study. The trial will be registered in the ClinicalTrials.gov clinical trials registry.

Budget

No funding. The project was carried out using research equipment and materials from the Department of Medical Biology, Poznań University of Physical Education.

Informed consent forms

(Polish version)

Dane osobowe pacjenta

Miejsce i data

………………………………

………………………………….

………………………………

………………………………

Oświadczenie o wyrażeniu zgody na osobiste uczestnictwo w badaniu naukowym pt.: „Wpływ terapii manualnej na subiektywne i obiektywne parametry dotyczące funkcjonowania kręgosłupa u osób z przewlekłym zespołem bólowym lędźwiowego odcinka kręgosłupa"

Wyrażam zgodę na uczestnictwo w badaniu oraz na anonimowe przetwarzanie uzyskanych danych w celach naukowych, włącznie z opublikowaniem ich w czasopiśmie naukowym. Zapoznałam/em się z informacją dotyczącą celu i przebiegu badania. Zostałam/em poinformowana/y o możliwości zadawania pytań prowadzącemu eksperyment i otrzymania odpowiedzi na te pytania oraz o możliwości odstąpienia od udziału w eksperymencie medycznym w każdym jego stadium.

…………………………………..

Podpis pacjenta

(English version)

Patient's personal data

…………………………………

…………………………………

…………………………………

Place and date

………………………………….

Statement of consent to personal participation in a scientific study entitled: "The influence of manual therapy on subjective and objective parameters of spine functioning in people with chronic low back pain"

I agree to participate in the study and to anonymously process the data obtained for scientific purposes, including their publication in a scientific journal. I have read the information regarding the purpose and course of the study. I have been informed about the possibility of asking questions to the experimenter and receiving answers to these questions, and about the possibility of withdrawing from participation in the medical experiment at any stage.

…………………………………..

Podpis pacjenta

Curriculum Vitae of main investigator

Nazwisko i imię: Wendt Michał

Nationality: polish

E-mail: wendt.m@interia.pl, wendt@awf.poznan.pl

Education:

- 15.12.2015 - Obtaining a doctoral degree in health sciences at the Faculty of Physical Education, Sport and Rehabilitation at the Poznań University of Physical Education
- 2010 - 2014 - Doctoral studies at the Faculty of Physical Education, Sport and Rehabilitation at the Poznań University of Physical Education
- 2004 – 2009 - Poznań University of Physical Education, professional title: Master of Physiotherapy

Experience:

- 1.10.2016 – till today - Assistant professor at the Department of Medical Biology at the Poznań University of Physical Education, (Królowej Jadwigi 27/39, 61-871 Poznań)
- 01.04.2009 – till today – Physiotherapist, NZOZ Rehabilitation Cabinets "Antidotum" (Stęszew 62-060, Reja 2/4)

Professional courses:

International Academy of Orthopedic Medicine IAOM (2016), Functional physiotherapy of patients with focal brain damage (2015), Diagnosis and treatment of pain syndromes of the spine and limbs using the McKenzie method (2015), Therapy of myofascial trigger points with palpation of the musculoskeletal system (2011), Deep tissue massage (2009), Classic massage course (2006).

Author of numerous publications in the field of physiotherapy in various peer-reviewed scientific journals.
